# Nobiletin Ameliorates Skeletal Muscle Performance in D‐Galactose‐Induced Aging Mice by Boosting Aerobic Metabolism

**DOI:** 10.1002/fsn3.71416

**Published:** 2026-01-09

**Authors:** Huihui Wang, Yangguang Zhang, Yijia Zhang, Xintong Wang, Yixuan Li, Fazheng Ren, Yanan Sun

**Affiliations:** ^1^ Beijing Advanced Innovation Center for Food Nutrition and Human Health, Department of Nutrition and Health China Agricultural University Beijing China; ^2^ State Key Laboratory of Food Nutrition and Safety, College of Food Science and Engineering Tianjin University of Science and Technology Tianjin China; ^3^ Key Laboratory of Functional Dairy, Co‐Constructed by Ministry of Education and Beijing Municipality, College of Food Science and Nutritional Engineering China Agricultural University Beijing China

**Keywords:** aerobic metabolism, mitochondria, nobiletin, sarcopenia, skeletal muscle function

## Abstract

The decrease in muscle performance during aging will not only lead to challenges in movement but also increase the risk of fractures, diabetes, and other diseases, which seriously impacts the overall quality of life in the elderly. In the present study, we used D‐galactose (D‐gal)‐induced 8‐week C57BL/6J mice to establish a sarcopenia model and to explore the effects of Nobiletin (Nob), a naturally occurring small molecule derived from orange peel, on skeletal muscle function. Our findings demonstrated that Nob significantly improved exercise endurance, grip strength, glucose tolerance, cold tolerance, and energy expenditure in D‐gal‐induced aging mice. Additionally, Nob ameliorated mitochondrial morphology and enhanced aerobic metabolism of myofibers in D‐gal‐induced aging mice, thereby providing increased energy for the body. Notably, Nob activated the SIRT1/PGC1α/Nrf2 pathway to enhance superoxide dismutase (SOD) activity and scavenge reactive oxygen species (ROS) in the skeletal muscle of D‐gal‐induced aging mice. In conclusion, Nob improved the metabolic capacity and function of skeletal muscle by augmenting its antioxidant capacity and optimizing mitochondrial function.

## Graphical abstract

Nob improved muscle function and prevented muscle atrophy in D‐gal‐induced aging mice. Nob enhanced the aerobic metabolism of myofibers in D‐gal‐induced aging mice. Nob activated the SIRT1/PGC1α/Nrf2 pathway, enhancing muscle metabolism and function.

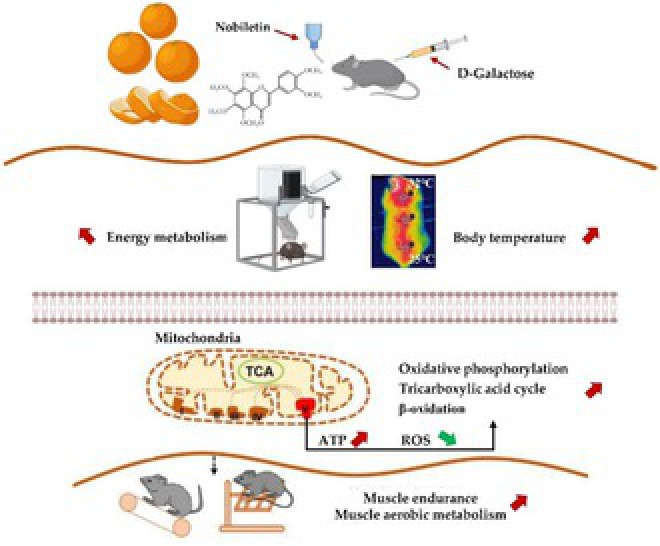

## 1 Introduction

Being the largest mitochondria‐rich organ in the human body, accounting for approximately 40% of healthy‐weight individual, skeletal muscle plays a crucial role in locomotion, thermogenesis, glucose metabolism, energy expenditure (Gan et al. 2018; Nohara et al. 2019; Pant et al. 2016; Sartori et al. 2021). The decrease in muscle performance during aging will not only result in challenges to movement, but also increase the possibility of fracture, diabetes and other diseases, which seriously impacts the overall quality of life of the elderly (Palla et al. 2021). It is reported that the skeletal muscle mass declined about 1%–2% per year after age 50, but the strength declined at 1.5%–5% per year (Abellan Van Kan 2009; Cruz‐Jentoft et al. 2019). As the global population aging increases, sarcopenia has become a prevalent geriatric condition and public health issue impacting about 15% of individuals aged 65 or above (Palla et al. 2021). Currently, there are no safe, effective, and low‐side‐effect medications approved for the treatment of sarcopenia. Although regular exercise combined with essential amino acid supplementation can help treat sarcopenia, exercise is not suitable for patients who are long‐term bedridden or have other clinical complications (Dao et al. 2020). Testosterone had been demonstrated to prevent age‐related loss of muscle strength and to ameliorate physical function (Srinivas‐Shankar et al. 2010; Storer et al. 2017); however, its clinical application is greatly limited by serious side effects (Grech et al. 2014). Even though some medications can improve the loss of muscle mass, most trials showed no significant amelioration of functional parameters (Furrer and Handschin 2019). For example, in a clinical trial, myostatin antibody (LY2495655) ameliorated muscle mass but not grip strength in the elderly (Becker et al. 2015). Skeletal muscle is still one of the most pharmacologically under‐treated organs. Therefore, there is an urgent need to identify safe, effective, and affordable drugs to improve skeletal muscle function or prevent muscle atrophy.

Nobiletin (Nob) is a natural flavonoid compound with six methoxy groups mainly found in the pericarp of citrus fruits and is well known for its antioxidative and anti‐inflammatory properties (Nohara et al. 2019; Wang et al. 2020). Nohara et al. found that Nob could increase ATP production and reduce reactive oxygen species (ROS) levels by optimizing mitochondrial function in skeletal muscle (Nohara et al. 2019). Wang et al. found that Nob significantly improved the behavioral deficits and neuroinflammation caused by lipopolysaccharides‐induced rats, and that Nob's neuroprotective and anti‐depressant effects mainly depend on its promotion of autophagy and inhibition of nucleotide‐binding oligomerization domain‐like receptor 3 (NLRP3) inflammasome activation (Wang et al. 2020). Our previous research had found that Nob increased protein synthesis, inhibited degradation pathways, and inflammatory cytokines to combat the loss of skeletal muscle mass during aging (Wang et al. 2023). Additionally, our other study had revealed that Nob not only improved mitochondrial function but also augmented autophagy function and protein synthesis pathways, thereby inhibiting skeletal muscle atrophy (Wang et al. 2022). Previous studies had suggested that enhancing the aerobic metabolism of skeletal muscle could significantly improve muscle function (Lv et al. 2023). Given the multiple known effects of Nob, such as its anti‐inflammatory and antioxidant properties, improvement of mitochondrial function, and maintenance of metabolic homeostasis, we were intrigued whether Nob could improve skeletal muscle function and prevent muscle atrophy by enhancing aerobic metabolic capacity and conducted further investigations.

In this study, an experimental model of skeletal muscle aging was established by D‐galactose (D‐gal) to gain insight into the effects of Nob on exercise capacity, aerobic metabolism level and its potential intrinsic mechanisms. Our findings will provide potential strategies for Nob to improve muscle function and treat muscle atrophy.

## 2 Materials and Methods

### 2.1 Main Reagents

Nobiletin (Nob) was obtained from MedChemExpress (Shanghai, China) with a catalog number of HY‐N0155 and a batch number of 151014, exhibiting a purity of 99.79%. SIRT‐1 inhibitor (EX‐527, HY‐15452) was obtained from MedChemExpress (Shanghai, China). D‐galactose (D‐gal, G5388) was sourced from Sigma‐Aldrich (St. Louis, MO, USA). The cDNA synthesis kit (G592) was purchased from abm (Vancouver, Canada). TRIzol (15596018CN) and PowerUp SYBR Green (A25742) were obtained from Thermo Fisher Scientific (Waltham, MA, USA). The Enhanced ATP Assay Kit (S0027), Lipid Peroxidation MDA Assay Kit (S0131M) and Total Superoxide Dismutase Assay Kit with WST‐8 (S0101M) were sourced from Beyotime Biotechnology (Shanghai, China). The mitochondrial respiratory chain complex IV activity assay kit (bc0945) was obtained from Beijing Solarbio Technology Co. Ltd. (Beijing, China). The PGC‐1α monoclonal antibody (66369‐1‐Ig), NRF2 Rabbit mAb (16396‐1‐AP), SIRT1 polyclonal antibody (13161‐1‐AP), MFN1 Polyclonal antibody (13798‐1‐AP), OPA1 Polyclonal antibody (27733‐1‐AP), and Beta Tubulin Polyclonal antibody (10094‐1‐AP) were purchased from Proteintech (Wuhan, China). The Mitofusin‐2 (D2D10) Rabbit mAb (9482) and DRP1 (4E11B11) Mouse mAb (14647) were obtained from Cell Signaling Technology. Mitochondrial Complex IV/Cytochrome C Oxidase Activity Assay Kit (bc0945) was obtained from Solarbio Technology Company (Beijing, China).

### 2.2 Mice Experiments

Forty‐five male C57BL/6J mice (body weight 25 ± 2 g, 8 weeks of age) were obtained from Beijing HFK Bioscience Co. LTD (Beijing, China). All mice were reared under pathogen‐free conditions at 25°C ± 1°C, 55%–60% relative humidity, a 12‐h light/dark cycle, and free access to food and water. All animal experimental procedures were in accordance with the Guide for the Care and Use of Laboratory Animals published by the National Institutes of Health and the Department of Public Health. In addition, the animal care and experimental procedures were reviewed and approved by the Ethics Committee for Animal Experiments of China Agricultural University (Approval No. AW60212202‐5‐1).

All mice were assigned ear tags numbered from 1 to 45, and after a one‐week acclimation period, they were randomly allocated to control group (CK group), model group (D‐gal group), and model + intervention group (D‐gal + Nob group) using a computer‐generated randomization sequence. After grouping, the body weights of the mice were measured to ensure that there were no statistically significant differences in baseline body weight between the groups. A model of sarcopenia was established in 8‐week‐old male C57BL/6J mice by subcutaneous injection of D‐gal for 10 weeks according to the method of Wang et al. (2023). Throughout the experimental period, the caloric intake and body weight of the mice were monitored weekly, and the mice received oral gavage and subcutaneous injection treatments between 8:00 AM and 10:00 AM daily. The mice in the D‐gal group were injected with 500 mg/kg D‐gal once a day. In contrast, the CK group was given the same volume of 0.9% physiological saline. The D‐gal + Nob group mice were orally administered Nob at a dose of 100 mg/kg/day after injection of D‐gal, and the CK and model groups mice were given the same volume of 0.9% physiological saline (containing DMSO).

### 2.3 Cell Culture and Treatment

C2C12 myoblasts were obtained from the Institute of Basic Medicine, Chinese Academy of Medical Sciences. The cells were incubated in DMEM medium supplemented with 10% fetal bovine serum (FBS) and 1% penicillin/streptomycin (P/S) in a humidified incubator at 37°C with 5% CO_2_. Upon reaching 80%–90% confluence, the cells were switched to differentiation medium containing 2% horse serum and 1% P/S to promote myotube formation. The myotubes were then treated with a SIRT‐1 inhibitor (EX‐527) for 48 h (Guo et al. 2024). Subsequently, the myotubes were exposed to differentiation medium containing 20 g/L D‐gal for 48 h to induce cellular senescence, with or without the intervention of 20 μM NOB (Guo et al. 2024).

### 2.4 Muscle Fatigue Test

Muscle endurance was performed using a motorized treadmill as described by Nohara et al. (2019). The mice were adapted for 3 days prior to the formal experiments. The running speed was increased at a rate of 1 m/min every 5 min from an initial speed of 6 m/min. The run was considered over when the mice were unable to return to the treadmill or stay on the shock grid for more than 10 s. Then, the total time traveled by the running mice was calculated.

### 2.5 Grip Strength Test

According to the method of Lv et al. (2023), grip strength was conducted using a grip test meter. The grip test meter was placed on a horizontal stage. Once the mice grasped the horizontal bar of the sensing platform (the grip strength meter) with all four limbs, their tails were gently pulled horizontally backward until they released their grip of the bar. The grip strength meter automatically recorded the maximum grip force exerted. Each mouse underwent five repeated measurements, and the highest recorded value, measured in grams (“g”), was selected as the mouse's absolute grip strength and then averaged.

### 2.6 Intraperitoneal Glucose Tolerance Test

The intraperitoneal glucose tolerance test was conducted as follows (Ouyang et al. 2025). All mice were fasted for at least 13 h with free access to water before the formal experiments. Prior to the start of the experiments, the mice were weighed, and fasting blood glucose levels were recorded at 0 min. Subsequently, glucose was administered via intraperitoneal injection at a dose of 2 g/kg body weight. Blood glucose concentrations were then measured at 15, 30, 60, 90, and 120 min after glucose administration. A blood glucose concentration‐time curve was plotted with time (minutes) on the *x*‐axis and blood glucose concentration (mmol/L) on the *y*‐axis. The area under the curve (AUC) was calculated for the blood glucose concentration‐time curve.

### 2.7 Body Temperature

A single mouse was placed in a single cage and acute cold exposure at 4°C for 1 h and its body surface temperature and heat image were immediately measured with an infrared camera (FLIR T420, FLIR systems AB, Sweden), referring to the method of Chen et al. (2021).

### 2.8 Energy Metabolism

According to the methods of Akakabe et al. and Shi et al. metabolic assessment of mice required a 4‐day cycle (Akakabe et al. 2013; Shi et al. 2018). On the first day, mice were placed in metabolic cages for adaptation; data collected during this acclimation period was excluded from statistical analysis. Then, measurements were taken continuously for the next 3 days to obtain experimental data. Before placing the mice in the metabolic cages, their weights were recorded, and enough food and water were provided. Prior to measurements, the silica gel desiccant and NH_3_ filter in the equipment were checked, and the metabolic cage apparatus was calibrated. Parameters were set according to experimental requirements, and mice to be tested were placed in respective testing cages based on ear tag numbers. The measurement process began by initiating the program. Daily checks were conducted at set times to ensure proper functioning of the equipment, monitor the mice's condition, and confirm adequate food and water supplies.

### 2.9 Transmission Electron Micrographs (TEMs) of Muscle Tissues

Tibialis anterior muscle samples measuring 3 × 1 × 1 mm were cut along the longitudinal axis of the myofibers and fixed overnight in 2.5% glutaraldehyde solution at 4°C, following the method described by Zepeda et al. (2014). A blinded investigator examined the ultrastructure of intermyofibrillar mitochondria using a transmission electron microscope (Hitachi H7500 TEM, Japan) at various magnification levels. Muscle tissues from three mice were included in each experimental group. For each sample, six electron micrographs were randomly captured at each magnification to evaluate mitochondrial size, number, and density.

Analysis of mitochondrial size, number, and cristae abundance was performed using Fiji/ImageJ software. Morphometric analysis included at least six images per experimental group (Ding et al. 2022). Cristae were manually identified in high‐resolution, high‐magnification TEM images and outlined using a stylus in ImageJ to quantify the length of cristae (Lu et al. 2022). Cristae abundance was expressed as the total cristae length per mitochondrial area (μm/μm^2^).

### 2.10 RNA Isolation and Quantitative Real‐Time PCR (qRT‐PCR)

Total RNA was extracted using the TRIzol method. The extracted RNA was then converted to cDNA using a cDNA synthesis kit, following the manufacturer's instructions. Subsequently, quantitative real‐time PCR (qRT‐PCR) was performed using the PowerUp SYBR Green kit. The primers utilized in this study are listed in Table 1. Relative gene expression levels were calculated using the 2^−ΔΔCt^ method (Yang et al. 2021), normalizing to the housekeeping gene *GAPDH*. The control group served as the calibrator sample. The formula used was:

$$\text{Fold Change}=2^{–\left[\mathrm{\Delta Ct}\left(\text{treated}\right)–\mathrm{\Delta Ct}\left(\text{control}\right)\right]}$$

where ΔCt = Ct(target) – Ct(reference).

**TABLE 1:** Primer sequences of RT‐qPCR.

Gene name	Primer sequence 5′→3′ (forward)	Primer sequence 5′→3′ (reverse)
*Mb*	CTGTTTAAGACTCACCCTGAGAC	GGTGCAACCATGCTTCTTCA
*Atp5b*	GAGGGATTACCACCCATCCT	CATGATTCTGCCCAAGGTCT
*Cycs*	GGAAGACCCTAATCTAGTCCCG	GTTGGGGCATCGCTGACTC
*Cox5b*	CTGTTTAAGACTCACCCTGAGAC	CACCCTGACATAGACAGTGAAAG
*Cox2*	CACCCTGACATAGACAGTGAAAG	CTGGGTCACGTTGGATGAGG
*Cpt1b*	TGGGACTGGTCGATTGCATC	CAGGGTTTGTCGGAAGAAGAAAA
*Ucp3*	ACTCCAGCGTCGCCATCAGGATTCT	TAAACAGGTGAGACTCCAGCAACTT
*Pdk4*	CCGCTGTCCATGAAGCA	GCAGAAAAGCAAAGGACGTT
*Ndufa5*	ATGGCGGGCTTGCTGAAAA	GCTGCATGTTTAGGAAAGTGCTT
*Sdhb*	AATTTGCCATTTACCGATGGGA	AGCATCCAACACCATAGGTCC
*Uqcrq*	CCTACAGCTTGTCGCCCTTT	GATCAGGTAGACCACTACAAACG
*Uqcc3*	TTGCTGGTGCGAGTCCTTAG	GTGTCCGCTCCAACAGTCT
*Idh2*	GGAGAAGCCGGTAGTGGAGAT	GGTCTGGTCACGGTTTGGAA
*Mdh1*	TTCTGGACGGTGTCCTGATG	TTTCACATTGGCTTTCAGTAGGT
*Ogdhl*	GGATGCAGACCTAGATTCCTTTG	GGCAGCCGGAACTCCTTAT
*Cox7a*	GCTCTGGTCCGGTCTTTTAGC	GTACTGGGAGGTCATTGTCGG
*GAPDH*	TGGCCTTCCGTGTTCCTAC	GAGGCTGTGAAGTCGCA

### 2.11 Western Blot Analysis

According to the method of Kang et al., western blotting was used to determine the expression of SIRT1, PGC1α, and Nrf2 proteins (Kang et al. 2018). The muscle tissues were lysed in a tissue lysis buffer containing protease inhibitors, and protein quantification was performed using the micro‐BCA protein assay kit. Equal amounts (50 μg) of protein were separated by sodium dodecyl sulfate‐polyacrylamide gel electrophoresis on 10%–12% gels. Subsequently, the separated proteins were transferred onto PVDF membranes. After blocking, PVDF membranes were incubated overnight at 4°C with specific antibodies against Nrf2, PGC1α, and SIRT1. Next, the membranes were probed with horseradish peroxidase‐conjugated secondary antibodies at room temperature for 2 h. Protein bands were visualized and quantified using an ECL imaging system. The relative expression of target proteins was normalized to the housekeeping protein GAPDH.

### 2.12 Mitochondrial Complex IV Activity Assays

Weigh approximately 100 mg of gastrocnemius muscle, chop it into small pieces, and add 1.0 mL of extraction buffer to homogenize. Then, centrifuge at 600 g for 10 min at 4°C, transfer the supernatant to another tube, and centrifuge again at 11000 g for 15 min to obtain the cytosolic extract, which can be used to measure the leakage of Complex IV from the mitochondria. Add 400 μL of extraction buffer to the pellet and perform ultrasonic disruption (200 W, sonicate for 5 s, with a 10‐s interval, repeated 15 times) for the measurement of Complex IV enzyme activity and protein content.

### 2.13 Measurement of ATP

Total ATP in muscle was measured according to the Enhanced ATP Assay Kit instructions. Fifty milligrams of tibialis anterior muscle was taken, and 500 μL of lysis buffer was added. The tissue was homogenized thoroughly using a glass homogenizer to ensure complete lysis. After lysis, the mixture was centrifuged at 4°C, and the supernatant was collected. Then, 100 μL of ATP detection working solution was added to the assay wells to deplete background ATP, followed by the addition of 20 μL of the supernatant. The mixture was quickly mixed, and RLU values were measured using a luminometer. Total ATP levels were calculated on the basis of the luminescence signals accordingly.

### 2.14 Measurement of ROS

100 mg of tibialis anterior muscle was taken and homogenized in 0.5 mL of PBS buffer on ice. The mixture was centrifuged at 4°C for 15 min (2000 g), and the supernatant was collected. The protein concentration of the supernatant was determined using a BCA assay kit and diluted to 0.1 mg/mL. 2 mL of the diluted supernatant was mixed with 2 mL of PBS buffer (containing 5 μmol/L DCFH‐DA), and the fluorescence value before incubation was quickly measured. After incubating at 37°C for 15 min, the fluorescence value after incubation was measured. The measurement conditions were as follows: the emission wavelength was 420 nm, and the excitation wavelength was 325 nm (slit width 5 nm). Relative ROS content = (fluorescence value after incubation − fluorescence value before incubation)/incubation time × protein concentration.

### 2.15 Measurement of MDA

50 mg of tibialis anterior muscle was taken, and 500 μL RIPA lysis buffer containing 100 mM PMSF was added along with 3–4 glass beads; the mixture was homogenized at 8000 rpm in a tissue homogenizer under low temperature conditions. It was then centrifuged at 12000 × g at 4°C, the supernatant was collected, and its protein concentration was determined using a BCA assay kit. A standard curve was prepared, and samples were mixed with working solution. After mixing, the tubes were heated in a 100°C water bath, then cooled to room temperature and centrifuged at 1000 × g at room temperature. 200 μL of supernatant was taken into a 96‐well plate, and the absorbance was measured at 532 nm using a microplate reader.

### 2.16 Measurement of SOD

50 mg of tibialis anterior muscle was taken, 500 μL of SOD sample preparation solution was added, and the tissue was homogenized using a glass homogenizer with thorough homogenization to ensure complete lysis; after lysis, the mixture was centrifuged at 4°C and the supernatant was collected. Appropriate amounts of WST‐8/enzyme working solution and reaction initiation solution were prepared. The test samples and other solutions were added in sequence, and after adding the reaction initiation working solution, the mixture was thoroughly mixed, incubated at 37°C for 30 min, and then the absorbance was measured at 450 nm using a microplate reader.

### 2.17 Statistical Analysis

All findings are presented in the form of average plus or minus SEM. Statistical significance was determined by one‐way ANOVA with Tukey post hoc tests for multiple‐group comparisons. All figures were plotted with GraphPad Prism 8.

## 3 Results

### 3.1 Effect of Nob on Skeletal Muscle Function in D‐Gal‐Induced Aging Mice

**FIGURE 1:**
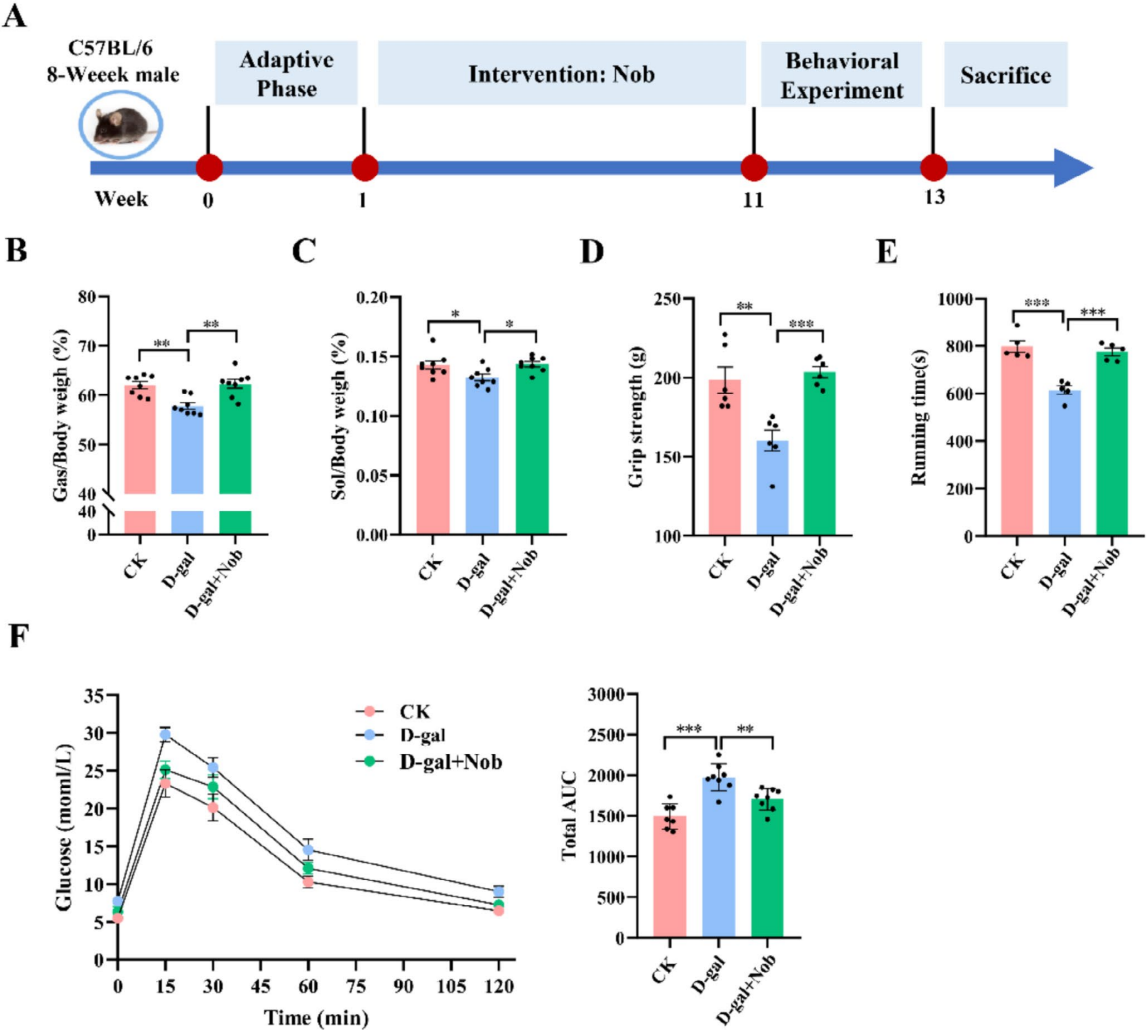
Nob improved skeletal muscle mass and function in D‐gal‐induced aging mice. (A) Experimental timeline. (B) Gastrocnemius (Gas)‐to‐body weight ratio was measured after 10 weeks of treatment, *n* = 8/group. (C) Soleus (Sol)‐to‐body weight ratio was measured after 10 weeks of treatment, *n* = 8/group. (D) Grip strength, *n* = 6/group. (E) Running time, *n* = 5/group. (F) Glucose tolerance test (GTT), and corresponding area under curve (AUC) of glucose concentration‐time were calculated (*n* = 7, 8, and 8/group for the CK, D‐gal, and D‐gal + Nob groups, respectively). *p < 0.05, **p < 0.01, ***p < 0.001.

To establish a sarcopenia mouse model, C57BL/6J mice were injected with D‐gal 10 weeks. Simultaneously, an equal volume of normal saline or Nob was administered daily by gavage (Figure 1A). The results indicated that D‐gal accelerated the loss of gastrocnemius (Gas) and soleus (Sol) muscles, leading to the model group exhibiting the lowest ratio of Gas to Sol. Following the nobiletin intervention, the D‐gal + Nob groups showed significant improvement in muscle (Figure 1B,C). Treadmill and grip strength assays demonstrated that D‐gal significantly decreased both grip strength and running time compared to the CK group (Figure 1D,E). In contrast, nobiletin treatment significantly improved grip strength and running time in D‐gal‐induced aging mice, indicating that Nob enhanced muscle exercise capacity. Skeletal muscle is not only the primary site for glucose uptake and storage but also accounts for 80% of glucose metabolism in the body (Sun et al. 2020). Glucose tolerance is a key indicator of insulin sensitivity in regulating blood glucose levels. The area under the curve (AUC) of the glucose concentration‐time profile is inversely related to glucose tolerance (Ouyang et al. 2025). In the D‐gal‐induced aging model group, the AUC of the glucose concentration‐time curve was significantly impaired compared to the CK group; however, it partially recovered to normal levels following nobiletin intervention (Figure 1D). Therefore, we next conducted glucose tolerance assays following the methods described by Nohara et al. (2019). In summary, although aging diminishes skeletal muscle mass and function, nobiletin effectively increased muscle mass, restored exercise capacity and improved glucose homeostasis in D‐gal‐induced aged mice.

### 3.2 Effect of Nob on Skeletal Muscle Thermogenesis and Metabolism in D‐Gal‐Induced Aging Mice

**FIGURE 2:**
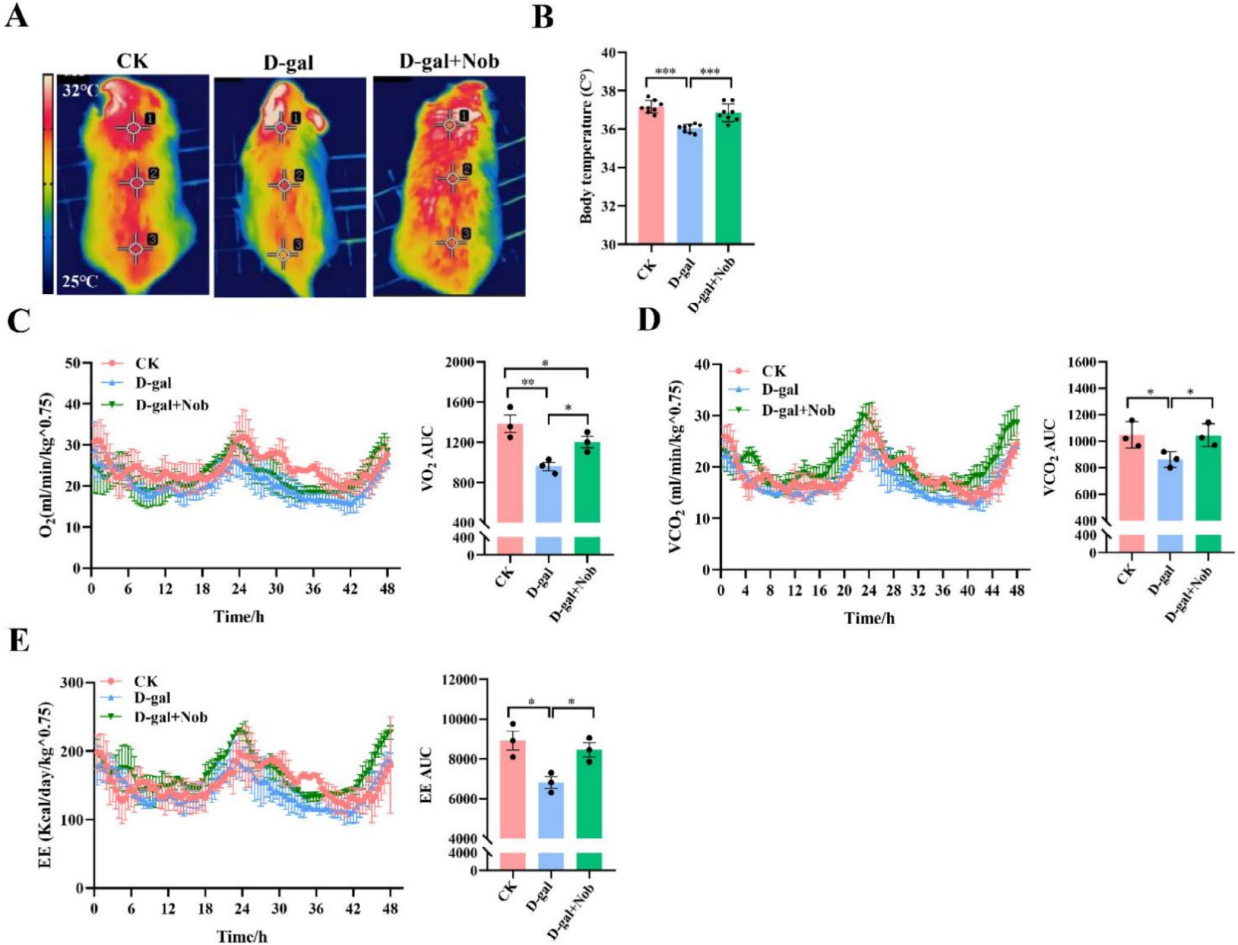
Nob ameliorated skeletal muscle adaptive thermogenesis and metabolism in D‐gal‐induced aging mice. (A) Representative infrared thermal image map, the gradation of the color of the temperature scale on the left, from blue to red, represents a gradual increase in temperature from 25°C to 32°C, *n* = 8/group. (B) Surface temperature, *n* = 8/group. (C) VO_2_ consumption and VO_2_ consumption AUC, *n* = 3/group. (D) VCO_2_ production and VCO_2_ production AUC, *n* = 3/group. (E) Energy expenditure (EE) and Energy expenditure AUC, *n* = 3/group. *p < 0.05, **p < 0.01, ***p < 0.001.

Skeletal muscle plays a crucial role in thermogenesis and temperature regulation (Pant et al. 2016). In thermoregulated animals, body temperature remains stable and does not fluctuate with changes in the external environment. When exposed to cold, skeletal muscles contract to generate heat, maintaining a constant body temperature When the body is acutely exposed to cold, skeletal muscles increase their contractions to produce heat, thus maintaining a constant body temperature (Pant et al. 2016). To assess the effect of Nob on the capacity for adaptive thermogenesis, we conducted a cold test. Infrared thermograms showed numerous red areas on the backs of mice in the CK group, while these areas were significantly reduced in the model group (Figure 2A). In contrast, Nob‐treated mice displayed increased red areas. Additionally, body temperature measurements indicated that D‐gal‐induced aging mice had markedly decreased compared with the CK group, whereas Nob treatment effectively restored body temperature (Figure 2B). Skeletal muscle contributes to 20%–30% of total resting oxygen uptake (Pant et al. 2016; Zurlo et al. 1990). Next, we measured in vivo energy metabolism using a metabolic chamber. With no significant changes in food intake (Figure S1A), Nob significantly increased O_2_ consumption, CO_2_ production, and energy expenditure in D‐gal‐induced mice (Figure 2E–G). Nob treatment also significantly reduced body fat content in D‐gal‐induced aging mice (Figure S1C). Although total energy expenditure in the Nob group increased, the body weight increased (Figure S1B). This may be due to Nob promoting fat breakdown while also increasing the proportion of lean body mass, similar to previous findings in high‐fat diet‐fed mice (Nohara et al. 2019).

### 3.3 Effect of Nob on the Aerobic Metabolism of Myofibers in D‐Gal‐Induced Aging Mice

**FIGURE 3:**
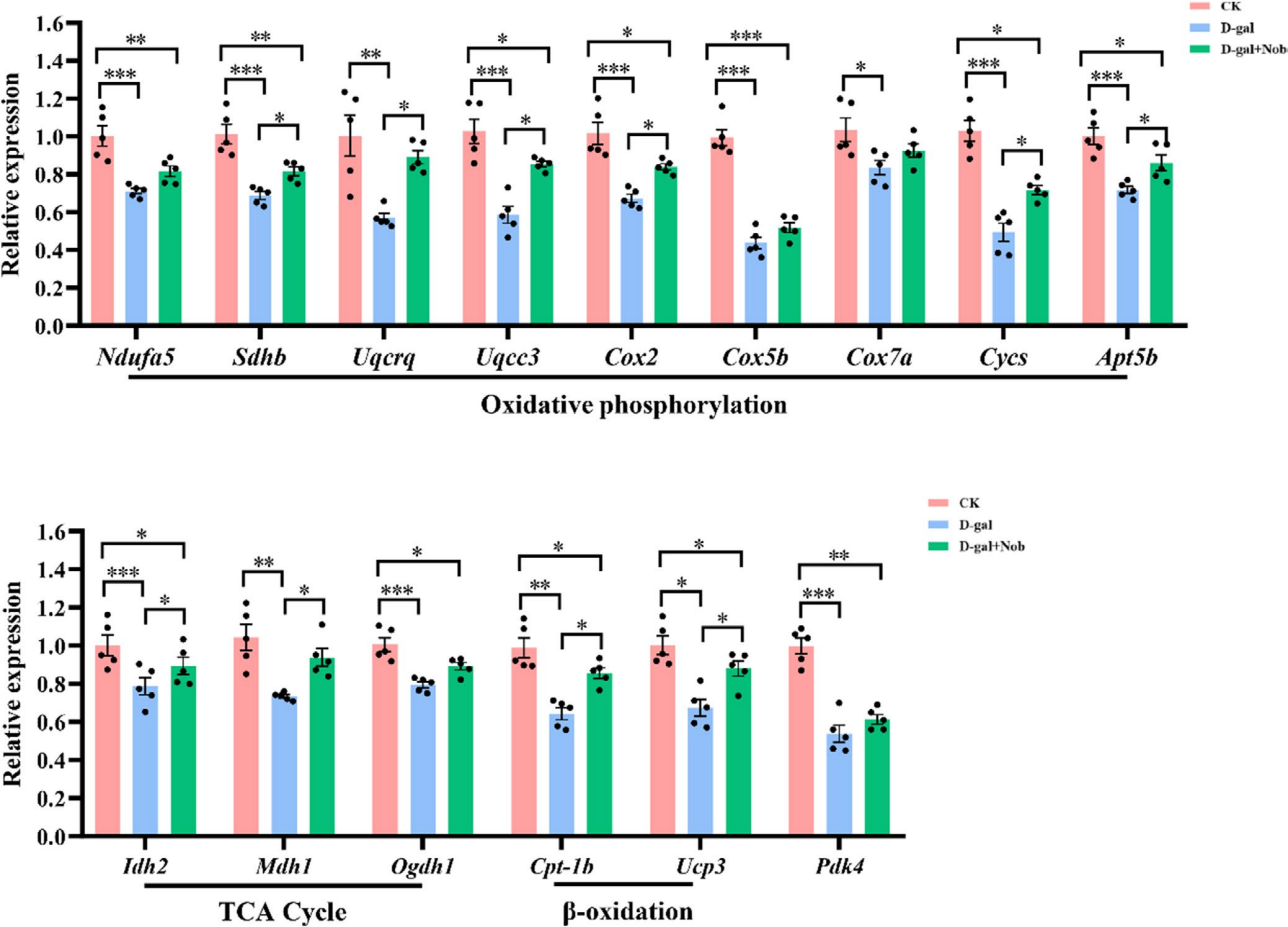
Nob enhanced the aerobic metabolism of myofibers in D‐gal‐induced aging mice. mRNA expression of related genes involved in oxidative phosphorylation, the TCA cycle, β‐oxidation, and glucose metabolism in D‐gal‐induced aging mice treated with vehicle or Nob, *n* = 5. *p < 0.05, **p < 0.01, ***p < 0.001.

The increased muscle endurance and metabolic capacity phenotype suggested that Nob likely influences energy metabolism in skeletal muscle in D‐gal‐induced aging mice. Therefore, we examined the expression of several metabolism‐related genes in these three groups of mice. Compared with the CK group, the D‐gal‐induced aging mice exhibited a significant reduction in the mRNA levels of oxidative phosphorylation‐related genes (*Ndufa5*, *Sdh*, *Uqcrq*, *Uqcc*, *Cox2*, *Cox5*, *Cox7a*, *Cyc*, and *Atp5b*) (Figure 3). However, Nob treatment partly upregulated the mRNA expression of oxidative phosphorylation‐related genes to normal levels, suggesting that Nob intervention greatly enhanced oxidative phosphorylation (OXPHOS) of myofibers of D‐gal‐induced aging mice. Likewise, whereas D‐gal reduced the mRNA expression of TCA cycle‐related genes (*Idh2*, *Mdh1*, and *Ogdh1*) compared with the CK group, Nob also partly restored these parameters to normal levels. Consistent with its effect on OXPHOS and the TCA cycle, Nob also greatly increased the mRNA expression of β‐oxidation‐related genes (*Cpt‐1* and *Ucp3*) to normal levels relative to the D‐gal group (Figure 3). Furthermore, the glucose metabolism‐related gene (*Pdk4*) was upregulated by Nob in the D‐gal‐induced aging mice (Figure 3). Taken together, these results indicate a robust efficacy of Nob to enhance oxidative metabolism parameters, which provided more energy for the body.

### 3.4 Effect of Nob on Mitochondrial Function in D‐Gal‐Induced Aging Mice

**FIGURE 4:**
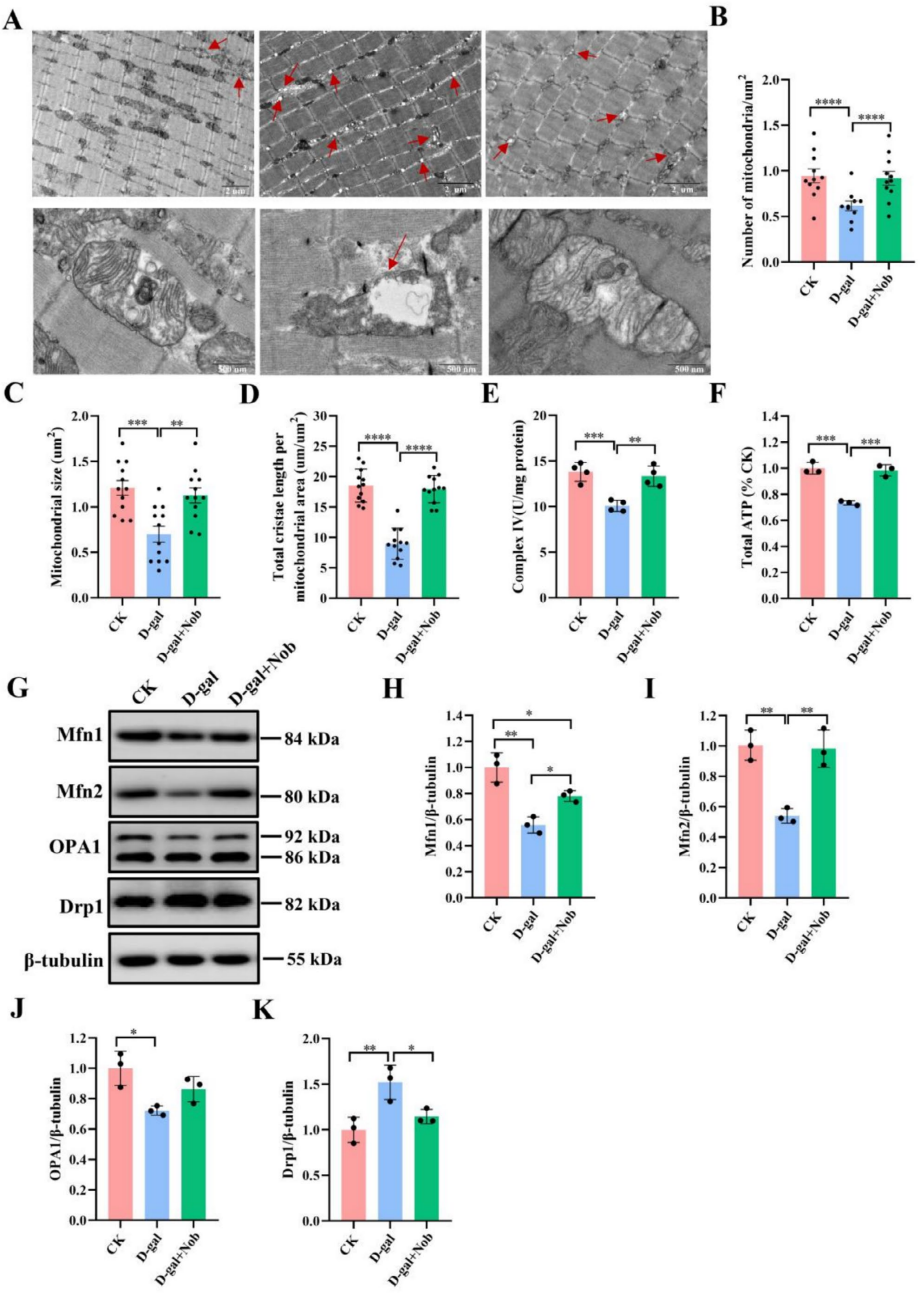
Nob ameliorated mitochondrial ultrastructure and function in D‐gal‐induced aging mice. (A) Mitochondrial ultrastructure, scale bar = 2 um or 500 μm, *n* = 3 mice/group. (B) Number of mitochondria. (C) Mitochondrial size. (D) Total cristae length per mitochondria area. (E) The activity of complex IV, *n* = 4 mice/group. (F) Total ATP production, *n* = 3. (G‐H) Western blot analysis of Mfn1, Mfn2, OPA1, Drp1 and β‐tubulin in D‐gal‐induced aging mice treated with vehicle and Nob, *n* = 3 mice/group. *p < 0.05, **p < 0.01, ***p < 0.001, ****p < 0.0001.

Mitochondria are vital for maintaining skeletal muscle function and myofiber homeostasis, as well as coordinating energy production through OXPHOS and fatty acid degradation (Gonzalez‐Freire et al. 2018; Xiao et al. 2021). In the present research, transmission electron microscopy was employed to examine changes in mitochondrial ultrastructure in skeletal muscle. Ultrastructural examination revealed that myofibers from the CK group exhibited mitochondria with clear cristae and a transparent electron‐dense matrix. In contrast, the model group displayed severe mitochondrial abnormalities, including vacuolization, partial loss of cristae, and a marked reduction in both the number and size of mitochondria (Figure 4A–D). However, Nob treatment markedly ameliorated these ultrastructural abnormalities, leading to a significant improvement in mitochondrial morphology, including a reduction in vacuolization and a more organized cristae structure (Figure 4A–D). On the other hand, while D‐gal‐induced mice showed markedly reduced mitochondrial respiratory chain complex IV activity and total ATP production, NOB treatment fully and significantly rescued these parameters (Figure 4E,F). Likewise, whereas D‐gal induction significantly decreased the expression of fusion proteins (Mfn1, Mfn2, and Opa1), NOB restored these proteins to varying degrees (Figure 4G–J). In addition, although D‐gal upregulated the expression of the fission protein Drp1, NOB treatment significantly downregulated its expression (Figure 4G,K).

### 3.5 Effect of Nob on the Antioxidant Capacity of Skeletal Muscle in D‐Gal‐Aging Mice

**FIGURE 5:**
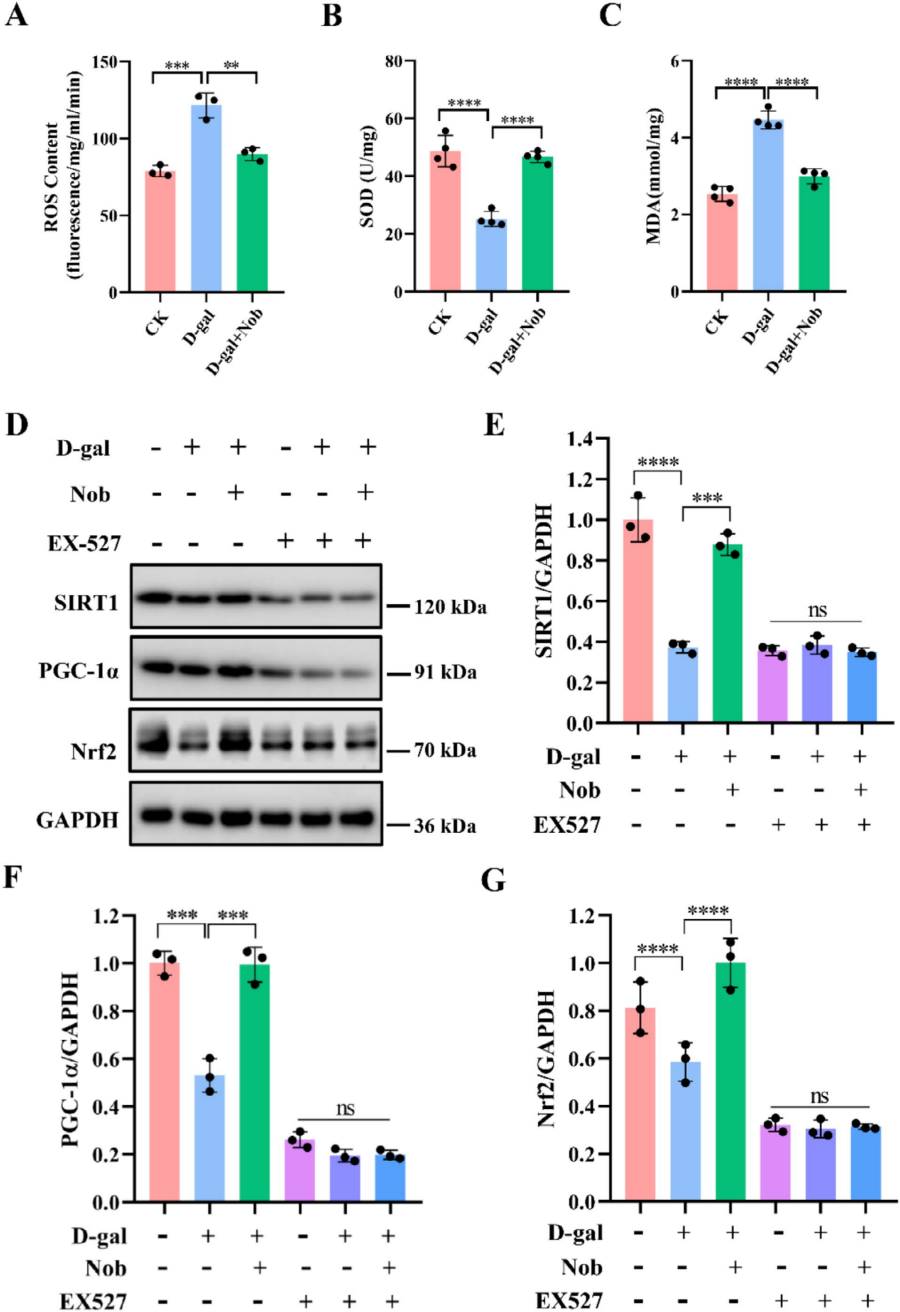
Nob improved the antioxidant capacity of skeletal muscle in D‐gal‐induced aging mice. (A) ROS content, *n* = 3 mice/group; (B) SOD activity, *n* = 4 mice/group; (C) MDA content, *n* = 4 mice/group; (E–G) Western blot analysis of SIRT1, PGC‐1α, Nrf2, and GAPDH in D‐gal‐induced C2C12 cells treated with vehicle and Nob, *n* = 3. **p < 0.01, ***p < 0.001, ****p < 0.0001.

Apart from ATP, mitochondrial respiration also produces ROS (Chapman et al. 2019; Nohara et al. 2019). If not controlled in time, increased ROS may damage intracellular lipids, proteins, and nucleic acids, ultimately leading to mitochondrial dysfunction and accelerated aging (Chapman et al. 2019; Davalli et al. 2016). Therefore, we examined the effect of Nob on the antioxidant capacity of skeletal muscle in D‐gal‐induced aging mice. Compared with the CK group, the D‐gal‐induced aging mice exhibited significantly increased ROS and malondialdehyde (MDA) production, while Nob treatment significantly reduced ROS and MDA production in D‐gal‐induced aging mice (Figure 5A,C). Likewise, whereas D‐gal induction reduced the activity of the antioxidant enzyme superoxide dismutase (SOD) activity compared with CK group, Nob fully restored this parameter to normal levels (Figure 5B). SIRT1 plays an crucial role in aging, apoptosis, and oxidative stress (Yang et al. 2021). The activation of SIRT1 can upregulate downstream PGC1α, leading to an antioxidant protective response that alleviates oxidative stress (Meng et al. 2020). Although D‐gal induction treatment reduced the expression of SIRT1, PGC1α and Nrf2 of skeletal muscle relative to the CK group, Nob treat significantly elevated the expression of SIRT1, PGC1α and Nrf2 (Figure 5E–G). Furthermore, EX527 significantly inhibited the beneficial effect of Nob. Together, these results provide evidence for Nob's ability to modulate antioxidant activity in skeletal muscle, suggesting that Nob may have protective effects against oxidative damage in aged mice. Together, these results provide evidence for the ability of Nob to modulate antioxidant activity in skeletal muscle, indicating that Nob may have protective effects on oxidative damage in aged mice.

## 4 Discussion

Sarcopenia is a syndrome of aging characterized by a decline in skeletal muscle mass and function, posing significant public health challenges that threaten the independence and quality of life of the elderly population (Cruz‐Jentoft et al. 2019). Current strategies for treating sarcopenia primarily include increasing protein intake (Chen et al. 2020), exercise and pharmacological interventions (Frampton et al. 2020; Robinson et al. 2018). However, in practical applications, these strategies often face limitations due to implementation difficulties or significant side effects. It is still necessary to find safe and effective foodborne substances to prevent and ameliorate sarcopenia.

The pathological mechanisms underlying sarcopenia are multifactorial, involving dysregulation of muscle protein synthesis and degradation, mitochondrial dysfunction (Picca and Calvani 2021), chronic inflammation, and oxidative stress (Kim et al. 2023; Meng and Yu 2010). Throughout the aging process, normal cells encounter pro‐senescent stimuli, including ROS and inflammation, resulting in elevated expression of senescence markers such as p21, p16, and p53. This accumulation of senescent cells leads to increased production of ROS and the senescence‐associated secretory phenotype (SASP), which in turn contributes to tissue dysfunction, including skeletal muscle atrophy (Khosla et al. 2020). Natural aging models are often time‐consuming and expensive, making accelerated aging models, such as the D‐gal‐induced aging model, more favorable due to their ease of application, shorter study duration, and higher survival rates (Azman and Zakaria 2019). The D‐gal model is commonly used due to its convenience and minimal side effects, effectively replicating the oxidative stress and inflammation linked to sarcopenia (Kou et al. 2017; Wang et al. 2022). Therefore, in this research, we used D‐gal induction to establish a sarcopenia model in order to assess the effect of Nob on skeletal muscle function and aerobic metabolic levels.

Nob is a natural substance with multiple biological functions and potential to treat sarcopenia (Bunbupha et al. 2021). In this study, Nob demonstrated significant efficacy in promoting healthy aging, as evidenced by improvements in skeletal muscle mass, exercise endurance, grip strength, energy expenditure, and cold tolerance. We found that Nob enhances the metabolic capacity and function of skeletal muscle by activating the SIRT1/PGC1α/Nrf2 pathway, increasing its antioxidant capacity, optimizing mitochondrial function, and improving metabolic efficiency. Skeletal muscle accounts for approximately 40% of total body mass and utilizes over 70% of metabolites during periods of intense physiological demand, such as exercise. Therefore, muscle plays a crucial role in regulating the overall metabolic rate, and any disruption to muscle health can significantly impact metabolism and overall function.

While this study offers preliminary evidence for Nob's potential in mitigating sarcopenia, several limitations should be considered. Firstly, the concomitant administration of Nob with D‐galactose in our design primarily supports its role in preventing rather than treating established muscle atrophy. This distinction is critical for clinical translation, as the mechanisms underlying preventive and therapeutic interventions may differ substantially. It is noteworthy that this strategy is supported by other aging intervention studies. For instance, research has shown that treatment with naringenin in 20–22 month‐old aged mice for 8 weeks did not significantly improve their skeletal muscle mass and function (Nohara et al. 2019). This result suggests that implementing a single treatment at the late stages of aging may have limited efficacy, further highlighting the potential value of nutritional prevention during the early stages of muscle decline or aging processes. Therefore, future studies should focus more on exploring the preventive effects of Nob in the early stages of aging or muscle wasting initiation. Furthermore, although the D‐gal‐induced aging model is well‐established, it does not fully recapitulate the complexity of natural aging in humans. The absence of validation in other models, such as naturally aged mice or genetic models of aging, limits the generalizability of our findings. To strengthen the translational relevance of Nob, subsequent research should incorporate multiple aging models, increase sample sizes, consider gender differences, and explore potential synergies with other interventions such as exercise or combined nutritional strategies. Finally, the dosage and safety profile of Nob require further systematic evaluation in clinical settings to ascertain its potential for human application.

## 5 Conclusions

In conclusion, this work demonstrated that Nob improved muscle endurance and prevented muscle atrophy by enhancing the aerobic metabolic capacity of myofibers. Furthermore, it identified that Nob enhanced the metabolic capacity and function of skeletal muscle by activating the SIRT1/PGC1α/Nrf2 pathway, augmenting its antioxidant capacity, optimizing mitochondrial function, and improving metabolic efficiency. Together, these findings provide a solid theoretical basis for future research and development of Nob as a medical food or pharmaceutical for the treatment of sarcopenia.

## Author Contributions

**Huihui Wang:** formal analysis (lead), investigation (lead), methodology (lead), resources (lead), software (lead), writing – original draft (lead). **Yangguang Zhang:** writing – original draft (supporting). **Yijia Zhang:** investigation (supporting), resources (supporting). **Xintong Wang:** writing – review and editing (equal). **Yixuan Li:** conceptualization (supporting), funding acquisition (supporting), project administration (supporting). **Fazheng Ren:** conceptualization (supporting), funding acquisition (supporting), project administration (supporting). **Yanan Sun:** conceptualization (lead), funding acquisition (lead), project administration (lead).

## Funding

This work was supported by the 9th China Association for Science and Technology Youth Talent Support Program (2023‐2026).

## Conflicts of Interest

The authors declare no conflicts of interest.

## Supporting information


**Data S1:** fsn371416‐sup‐0001‐supinfo.docx.

## Data Availability

The data that support the findings of this study are available from the corresponding author upon reasonable request.
