# Supplementary material for: Nobiletin Ameliorates Skeletal Muscle Performance in D‐Galactose‐Induced Aging Mice by Boosting Aerobic Metabolism
Source: Food Sci Nutr. 2026 Jan 9;14(1):e71416. doi: 10.1002/fsn3.71416 (PMC12784212; doi:10.1002/fsn3.71416)
Supplement: Supplementary file 1 — Data S1: fsn371416‐sup‐0001‐supinfo.docx. [file FSN3-14-e71416-s001.docx]

| 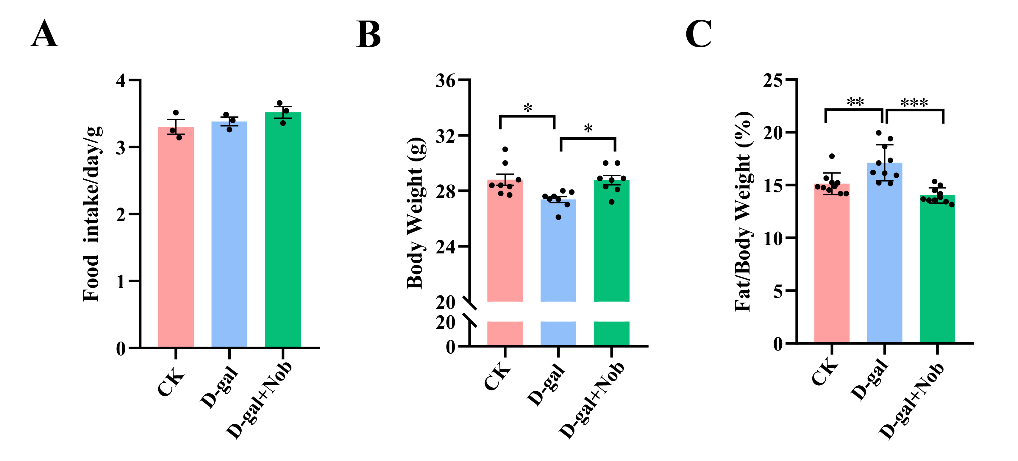 |
| --- |

Supplemental Figure. S1 Effects of Nob on food Intake, body weight, and fat-to-body weight ratio after 10 Weeks. (A) Food intake/day/g. (B) Body weight were measured after 10 weeks of treatment, n = 8/group. (C). Fat-to-body weight ratio were measured after 10 weeks of treatment, n = 10/group. **p* < 0.05, ***p* < 0.01, ****p* < 0.001.
